# Criteria in diagnosing nocturnal leg cramps: a systematic review

**DOI:** 10.1186/s12875-017-0600-x

**Published:** 2017-02-28

**Authors:** Joannes Hallegraeff, Mathieu de Greef, Wim Krijnen, Cees van der Schans

**Affiliations:** 1 Research Group Healthy Ageing, Allied Health Care and Nursing, Hanze University of Applied Sciences, Groningen, The Netherlands; 2 SOMT University Campus, Institute for Master Education in Musculoskeletal Therapies, Amersfoort, The Netherlands; 3 University of Groningen, University Medical Centre Groningen, Groningen, The Netherlands

**Keywords:** Cramps, Nocturnal, Diagnosis, Aged, Sleep-wake transition disorder, Restless legs syndrome

## Abstract

**Background:**

Up to 33% of the general population over 50 years of age are affected by nocturnal leg cramps. Currently there are no generally accepted clinical characteristics, which identify nocturnal leg cramps. This study aims to identify these clinical characteristics and to differentiate between them and the characteristics of restless leg syndrome and periodic limb disorder.

**Method:**

A systematic literature study was executed from December 2015 to May 2016. This study comprised of a systematic literature review of randomized clinical trials, observational studies on nocturnal and rest cramps of legs and other muscles, and other systematic and narrative reviews. Two researchers independently extracted literature data and analyzed this using a standardized reviewing protocol. Modified versions of the Cochrane Collaboration tools assessed the risk of bias. A Delphi study was conducted to assess agreement on the characteristics of nocturnal leg cramps.

**Results:**

After systematic and manual searches, eight randomized trials and ten observational studies were included. On the basis of these we identified seven diagnostic characteristics of nocturnal leg cramps: intense pain, period of duration from seconds to maximum 10 minutes, location in calf or foot, location seldom in thigh or hamstrings, persistent subsequent pain, sleep disruption and distress.

**Conclusion:**

The seven above characteristics will enhance recognition of the condition, and help clinicians make a clear distinction between NLC and other sleep-related musculoskeletal disorder among older adults.

## Background

Nocturnal Leg Cramps (NLC) is a musculoskeletal disorder characterized by suddenly occurring, episodic, persistently painful, involuntary contractions of the calf, hamstrings or foot muscles at night [1]. Up to 33% of the general population over 50 years of age have complaints related to NLC [2]. In 20% of these cases, cramps also occur during rest periods in the daytime [3]. Sleep disturbances, which may seriously affect well-being and quality of life, are common among patients with NLC [4, 5]. Symptoms, as well as prevalence and incidence, progress with advancing age [1, 6]. There is no consensus about aetiology of NLC, however it is suggested that shortened muscle length among older less physically active people is a risk factor [1]. Medical pathologies associated with NLC are chronic liver and renal failure (haemodialysis), vascular diseases, magnesium or calcium deficiency, dehydration and varicose veins [2, 7]. A pre-stretching protocol by physical therapists, as well as medical treatment blocking the medial branch of the deep peroneal nerve after lumbar surgery, may be effective in treating NLC [8, 9].

In contrast to the restless leg syndrome (RLS) and the idiopathic periodic limb movement disorder (PLMD), diagnosing NLC is hindered due to lack of a categorical definition of NLC. Moreover, different types of muscular cramps such as idiopathic, rest, leg, or pregnancy-related cramps are similar to NLC symptoms and are often confused in the literature [10].

Diagnostic criteria for RLS are clearly stated as follows: uncomfortable and unpleasant sensations in the legs, feet or arms associated with an urge to move; relief of symptoms by moving the affected limb; occurrence during rest in the evening or at night [11, 12]. The International Restless Leg Syndrome Study Group approved the validity of a rating scale for RLS, which reflects the severity of the discomfort [11]. Idiopathic PLMD symptoms include the repetitive jerking movements of the leg for approximately 20-30 seconds during sleep, with the complaints when awake being more intense than during sleep. PLMD can be classified into mild, moderate, and severe levels as measured by the Periodic Limb Movement Index. Additionally, both RLS and PLMD may co-exist [12]. No consensus has been reached regarding the diagnostic criteria for NLC, or how to differentiate them from the RLS and PLMD criteria [12]. Primarily based on the patient history, the diagnosis of NLC may be confused with RLS or PLMD [1].

Generally, nocturnal pain can be a symptom of a serious pathology such as Parkinson disease, cardiovascular and renal diseases, lumbar canal stenosis, osteroarthritis, peripheral neuropathy or cirrhosis. It is important to differentially diagnose NLC when is present as a nonspecific musculoskeletal disorder, or related to serious pathology.

This study focuses on strengthening the available criteria in order to prevent the misdiagnosis of NLC, for RLS or PLMD. The first aim of this literature review is to identify characteristics for diagnosing NLC. The second aim is to differentiate these diagnostic characteristics from other sleep-related disorders, such as RLS and PLMD, for application in clinical care.

## Method

A systematic review was done to identify diagnostic criteria of NLC. The methodology is specified in our PROSPERO-registered protocol (16467) and conforms to Preferred Reporting Items for Systematic Reviews and Meta-analyses (PRISMA) guidelines [13]. In order to differentiate between NLC, on the one hand, and RLS and PLMD, on the other, an additional Delphi methodology was used. In this study a focus group of 27 experts assessed the relevance of the diagnostic criteria.

### Sourcing information

An experienced librarian assisted in the development of a search strategy to identify recognized terminology. Four electronic databases were used including MEDLINE, Cinahl, EMBASE and PEDro (1990 to May 2016).

The search included all commonly used terms for NLC such as ‘cramps’, ‘muscle cramps’, ‘nocturnal leg cramps’, ‘leg cramps’, ‘night leg cramps’, ‘rest cramps’, ‘sleep-wake transition disorders/classification’, ‘aged’, ‘aging’, ‘elderly’, ‘senior’, ‘diagnosis’, ‘classification’, ‘epidemiology’, ‘rehabilitation’, ‘parasomnias’, ‘clinical trial’, ‘randomized controlled trial’, ‘observational study’, ‘clinical study’, ‘systematic review’, ‘meta-analysis’, ‘validation study’ or ‘letter’.

### Selection criteria

Inclusion criteria included randomised clinical trials, or observational studies reflecting NLC, muscle cramp, leg cramp, or rest cramp. The studies had to use the diagnostic criteria and classification in older adults aged over 50 years. A time frame spanning the previous 25 years.

Studies with non-English abstracts were excluded.

Two authors (JMH and MHGdG) independently extracted, screened, and reviewed all titles and abstracts of the retrieved articles. The articles were interpreted and classified into randomised clinical trials and observational studies. Reference lists of any recent reviews were hand searched in order to identify additional studies and help in excluding any duplicates.

### Data extraction

The characteristics, diagnostic features and population characteristics of the investigated populations were summarised and catalogued. Randomised clinical trials and observational studies were screened for descriptions of diagnostic terms or classification criteria for NLC during sleep among adults aged over 50. For each included study, descriptive data regarding the participants and diagnostic terms were extracted. A flowchart was made to show the process of the literature search [13].

### Quality assessment

Cochrane checklists for randomised clinical trials and observational studies were appraised using the methodological quality (risk of bias) of the included studies. To discuss any discrepancies between the two reviewers, consensus meetings were arranged. Complete agreement was reached after discussions with a third reviewer (CvdS) in all of the cases.

### Delphi sub-study

The Delphi methodology was performed to examine the relevance of the extracted diagnostic criteria found in the systematic review. A questionnaire with closed-ended questions on a five-point Likert scale (always – mostly – sometimes – never - not known) was presented to a focus group of experts. The questionnaire was developed based on the results of the literature search and comprised the following items: (1) Are you known with NLC; (2) NLC has a sudden onset; (3) NLC is only present at night; (4) Pain and/or intense pain is the main characteristic; (5) NLC duration varies from seconds to 10 minutes; (6) NLC location is thigh, calf or foot; (7) After reduction of NLC there will be pain afterwards; (8) NLC might be associated with sleep disruption; (9) NLC is associated with medication use / comorbidity; (10) NLC might be associated with distress. The designated criteria for inclusion were established by more than 50% of the respondents. Geriatric experts were randomly chosen on the basis of their expertise in geriatric health.

## Results

**Fig. 1:**
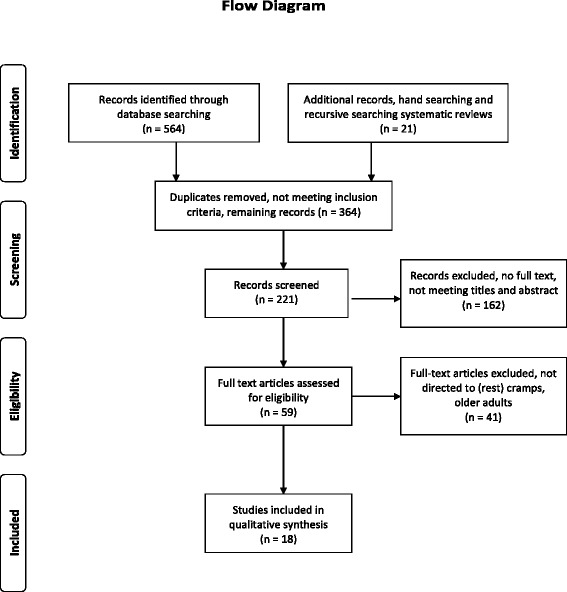
Flow diagram of the systematic review, modified from Moher et al., 2009 [13]

After completing the systematic search and manual searches of the reference lists of the systematic reviews and narrative reviews and after removing duplicates and records not meeting the inclusion criteria, in the screening a total of 221 papers were yielded of which 162 were irrelevant and had to be excluded due to not meeting titles and abstracts. This resulted in 59 records that were appropriate for further evaluation. Subsequently, 41 full-text articles were excluded because they did not describe diagnostic criteria of NLC in older adults. No primary studies with the focus on diagnosing were found. Eight randomised clinical trials and ten observational studies were eligible for analysing classification characteristics of NLC. Full consensus between MHG and WPK was reached regarding the included citations. Figure 1 presents the selection of the studies through the review process.

Two randomised clinical trials [14, 15] had high and unclear risk of bias due to a lack of internal validity, however the description of the included NLC patients was adequate.

The groups were treated equally in all studies, and the randomisation procedure was performed well, except in one instance. Among the observational studies two showed low risk of bias [4, 16]. In all studies, the populations were well defined.

See Tables 1 and 2 for risk of bias and Table 3 for the description of the study characteristics.

**Table 1:** Risk of bias of the included randomised clinical trials (9-item Cochrane checklists for randomised trials

	Randomisation	Concealed randomization	Blinded patients	Blinded treaters	Blinded Assessors	Groups comparable	Loss to follow up	Intention to treat	Groups treated equaly
Connolly 1992 [29] 6/9	+	-	+	+	-	-	+	+	+
Coppin 2005 [5] 8/9	+	+	-	+	+	+	+	+	+
Garrison 2011 [17] 9/9	+	+	+	+	+	+	+	+	+
Hallegraeff 2012 [9] 9/9	+	+	+	+	+	+	+	+	+
Jansen 1997 [18] 9/9	+	+	+	+	+	+	+	+	+
Roffe 2002 [19] 8/9	+	+	+	+	-	+	+	+	+
Serrao 2001 [14] 3/9	+	-	-	-	-	-	+	-	+
Young 1993 [15] 1/9	-	-	?	?	?	?	-	?	+

Coppin 2005 [5], Garrison 2011 [17], Hallegraeff 2012 [9], Jansen 1997 [18] and Roffe 2002 [19] showed low risk of bias

**Table 2:** Risk of bias of the included observational studies (9-item Cochrane checklist for observational studies)

	Groups well defined	Selection bias	Exposure	Outcome	Blinding	Follow-up	Loss to follow up	Confounding	Generalizability
Angeli 1996 [20] 6/9	+	-	+	+	-	+	+	-	+
Baskol 2004 [21] 6/9	+	+	+	+	-	?	?	+	+
Garrison 2015 [22] 9/9	+	+	+	+	+	+	+	+	+
Garrison 2012 [2] 6/9	+	-	-	+	+	+	+	-	+
Hawke 2013 [4] 9/9	+	+	+	+	+	+	+	+	+
Hawke 2013 [23] 9/9	+	+	+	+	+	+	+	+	+
Hirai 2000 [24] 4/9	+	-	+	+	-	?	?	-	+
Naylor 1994 [25] 5/9	+	+	+	-	+	?	?	?	+
Nishant 2014 [26] 5/9	+	-	+	+	-	+	-	-	+
Oboler 1991 [27] 4/9	+	-	+	+	-	?	?	-	+

Baskol 2004 [21], Garrison 2015 [22], Hawke 2013 [4] and Hawke 2013 [24] showed low risk of bias

**Table 3:** Characteristics of the included studies

Randomized clinical trials	Study objective Number of participants Age Male	Diagnostic criteria	Comorbidities associated with NLC and medication use
Connolly 1993 [29] 6/9	Efficacy of quinine *N* = 27 59 yrs. Male 100%	Nocturnal leg cramps. Aged > 50. Foot, lower part of leg, sometimes thigh. Sleep interruption.	Coronary artery disease, Peripheral vascular disease, Hypertension, Diabetes Mellitus *Medication:* diuretics
Coppin 2005 [5] 8/9	Effect of calf stretching *N* = 181 75 yrs. Male 46%	Nocturnal leg cramps, aged > 60, painful and involuntary. Muscle spasms. Disrupt sleep. Disruption. Most commonly in the leg, relief by stretching.	Renal dialysis, asthma and hypertension. *Medication:* diuretics, nifedipine, salbutamol and terbutaline
Garrison 2011 [17] 9/9	The effect of magnesium in individuals with leg cramps *N* = 46 69 yrs. Male 30%	Leg cramps, aged > 50, at rest (bed or night). Legs and feet. Painful muscle contractions.	Participants with comorbidities excluded
Hallegraeff 2012 [9] 9/9	Effect of pre sleep stretching *N* = 80 70 yrs. Male 50%	Nocturnal leg cramps, aged > 55, Suddenly, episodic. Involuntary. At rest or sleep. Calf, hamstrings or foot. Muscles are tender and hard. Intense painful. From seconds to minutes. Distress. Sleep disruption. Maximum ten minutes.	Varicose veins and arthritis. Physical inactivity and inadequate stretching and reduced muscle and tendon length. *Medication:* diuretics, lithium, steroids, morphine
Jansen 1997 [18] 9/9	Efficacy of hydro quinine in muscle cramps *N* = 102 54 yrs. Male 37%	Involuntary muscle contraction. Painful Sudden onset. Muscle hardening, maximum duration 10 minutes.	Not stated
Roffe 2002 [19] 8/9	The effect of magnesium in chronic non-pregnant individuals *N* = 36 63 yrs. Male 58%	Leg cramps. Painful contractions of any muscle group in the leg. Sudden onset. Successive improvement. Palpable hardening of the muscle. Distress.	Arthritis, peripheral vascular disease, varicose veins, ankle oedema
Serrao 2001 [14] 3/9	To evaluate the efficacy and safety of gabapentin in the treatment of muscle Cramps *N* = 30 54 yrs. 33%	Sudden, involuntary, painful contractions. Maximum of 10 minutes. Sleep disturbance.	Neuropathy, radiculopathy, Isaac’s syndrome, multiple sclerosis, Parkinson’s disease, vascular disease
Young 1993 [15] 1/9	The effect of naftidrofuryl in individuals with rest cramps *N* = 14 61 yrs. Male 64%	Rest cramps. Night-time cramps. Foot, calf muscles. Distress.	Not stated
Observational studies			
Angeli 1996 [20]	To define the features, prevalence, and pathophysiology of therapy for muscle and small muscles cramps in cirrhotic patients. *N* = 192 56 yrs. Male 65%	A-symmetric involuntary contractions or stiffness in calves and feet. At rest or at night	Cirrhosis, vascular occlusive disease, peripheral neuropathy, diabetes mellitus, severe renal failure and postphlebitic syndrome
Baskol 2004 [21] 6/9	The prevalence of muscle cramps in non-alcoholic cirrhosis patients. *N* = 65 52 yrs. Male 57%	Muscle cramps. Aged > 50. Involuntary. Painful, visible contraction. Sudden onset. At rest or sleep. From seconds to minutes. Affects quality of life. At least once per week. Sleep disruption.	Liver cirrhosis, diuretic, alcohol use, volume depletion, hyponatremia, haemodialysis, hypothyroidism, uraemia.
Garrison 2015 [22]	Seasonally variation of symptom burden of leg cramps in the general population. *N* = 31.339 69 yrs. Male 38%	Painful involuntary muscle cramps in the legs or feet during rest. It interrupts sleep.	Motor neuron disease, radiculopathy or hereditary cramp syndromes
Garrison 2012 [16] 6/9	Evaluating the association between diuretics, statins and long-acting β2 agonist’s use. *N* = 3492 69 yrs. Male 39%	Nocturnal leg cramps. Painful legs or feet. During rest or sleep	*Medication:* diuretics and long-acting β2 agonists
Hawke 2013 [4] 9/9	Impact of NLC on health related quality of life. *N* = 160 71 yrs. Male 41%	Nocturnal leg cramps. Pain afterwards. Sleep disruption. Aged >60 with sleep disruption. Reduced quality of life. Gradually lessens. Sudden, involuntary painful contraction. At night and rest. Relief by stretching.	Participants with comorbidities known to cause cramp excluded
Hawke 2013 [23] 9/9	Factors associated with night-time calf muscle cramps *N* = 160 71 yrs. Male 41%	Reduced strength dorsiflexion foot. Distress. Lesser quality of life. Interference of activities of daily living.	Hamstring tightness. Foot or leg coldness Participants with comorbidities known to cause cramp were excluded
Hirai 2000 [24] 4/9	NLC in general population and in patients with varicose veins *N* = 333 Age not stated Male 26%	Muscle cramps. Aged >50. Intense painful with sudden onset in calf, foot or thigh. Maximum duration 10 minutes. At least once per week.	Varicose veins
Naylor 1994 [25] 5/9	Prevalence, severity and correlation with vascular diseases *N* = 86 73 yrs. Male 44%	Rest cramps. Aged > 50. Distress. Less quality of life.	Peripheral vascular disease
Nishant 2014 [26] 5/9	Prevalence of nocturnal leg cramps in LSCS patients and in general population. *N* = 70 56 yrs. Male 53%	Nocturnal cramps. Painful. Acute and involuntary. At sleep or rest. Knee flexion test might be indicative for NLC.	Amyotrophic lateral sclerosis, poliomyelitis, peripheral neuropathy, lumbar spinal radiculopathy; metabolic disorders including diabetes, pregnancy, uremia, liver cirrhosis, and hyper- and hypothyroidism; acute extracellular volume depletion including excessive perspiration, hemodialysis, diarrhea, and diuretic therapy; hereditary disorder. Hypertension, hypocalcaemia, hypokalaemia, vascular diseases. *Medications:* diuretics, antidepressants, calcium blockers, statins, and steroid, nifedipine-blockers.
Oboler 1991 [27] 4/9	Prevalence and treatment regimens of NLC *N* = 262 60 yrs. Male 95%	Painful and involuntary in the calf with a visible palpable knot. At rest or sleep.	Arthritis, Peripheral vascular disease, Hypokalaemia, Coronary artery disease, Hypertension, Kidney disease, Stroke, Diabetes Mellitus, Hypocalcaemia.

In total 18 studies are included for analysing NLC characteristics: eight randomized clinical trials and ten observational studies

The total number of participants in the 18 included studies was 36,515 of which the study of Garrison et al. 2015 included 31,339 participants. Overall, 51% of the participants were male and mean age of participants was 64 years (range 51-75 years).

Comorbidities were categorized in five domains:

- Heart and vascular diseases: coronary artery disease, peripheral vascular disease, hypertension, varicose veins, ankle oedema, vascular occlusive disease and leg claudication.
- Kidney diseases: renal dialysis, haemodialysis, uraemia, hypocalcaemia and hypokalaemia.
- Neurological diseases: neuropathies, motor neuron disease, radiculopathy or hereditary cramp syndromes, neuromuscular or neurological diseases, peripheral neuropathy, Amyotrophic Lateral Sclerosis, poliomyelitis, lumbar spinal radiculopathy, lumbar canal stenosis and stroke.
- Musculoskeletal disorders: arthritis and myopathies.
- Metabolic disorders: Diabetes Mellitus, plasma electrolyte abnormalities hepatic, liver cirrhosis, postphlebitic syndrome volume depletion, hyponatremia, hypothyroidism, hyper- and hypothyroidism and acute extracellular volume depletion including excessive perspiration.

The analysis of 18 primary studies revealed twelve different diagnostic criteria used: ‘rest, sleep or night’ (*n* = 16); ‘painful’ (*n* = 12); ‘aged > 50’ (*n* = 8); ‘involuntary’ (*n* = 10); ‘sudden onset’ (*n* = 7; ‘posterior calf, foot or thigh’ (*n* = 8); ‘sleep disruption’ (*n* = 7); ‘persisting pain afterwards’ (*n* = 4); ‘duration from seconds to several minutes’ (*n* = 5); ‘distress’ (*n* = 4); ‘stiffness’ (*n* = 1) and ‘asymmetrical cramps’ (*n* = 1) [4, 9, 14–27].

### Clinical classification characteristics

After counting the number of times the criteria were described, and after comparing the twelve criteria to RLS and PLMD criteria, the following criteria were deemed not distinctive enough: ‘at rest or sleep’, ‘aged’, ‘involuntary’, ‘sudden onset’, ‘stiffness’ and ‘asymmetrical’. As a result, the following seven criteria remained in order to differentiate NLC from RLS and PLMD: ‘pain’, ‘intense pain’, ‘period of seconds to a maximum of 10 minutes’, ‘located in posterior calf or foot’, ‘subsequent pain’, ‘sleep disruption’ and ‘distress’. These seven classification characteristics differentiate from RLS and PLMD See Table 4.

**Table 4:** Involuntary musculoskeletal disorders at rest or nocturnal with sudden onset in elderly above 50

	Nocturnal leg cramps	Restless leg syndrome	Periodic Limb Movement Disorder
Pain	✓		
Intensely pain	✓		
From seconds to maximum 10 minutes	✓		
Calf or foot, seldom thigh	✓		
Persisting pain afterwards	✓		
Sleep disruption	✓		
Distress*	✓		
Irritating, burning, crawling sensations		✓	
In episodes		✓	
An urge to move		✓	
Reduction of symptoms by activity		✓	
No pain		✓	✓
Repeating and jerking movements			✓
Duration 20-30 seconds			✓

Reduced strength of dorsiflexion of ankle, foot and toes was also found in one study and can be associated with NLC [23]

The response rate of the geriatric clinicians in the focus group of the Delphi study was 52%, all with > 50% consensus. See Table 5

**Table 5:** Delphi study items

Delphi Study Items	Always	Mostly	Sometimes	Never	Not known
Are you known with NLC	30*	40	20	0	10**
• NLC has a sudden onset	33	56	11	0	0
NLC is only present at night	11	68	11	0	11
• Pain and / or intense pain is the main characteristic	10	80	0	0	10
• NLC duration varies from seconds to 10 minutes	10	80	0	0	10
• NLC location is thigh, calf or foot	33	45	11	0	11
• After reduction of NLC there will be pain afterwards	0	50	40	0	10
• NLC might be associated with sleep disruption	10	50	20	0	20
NLC is associated with medication use / comorbidity	0	11	67	0	22
• NLC might be associated with distress	10	10	60	10	10

Seven criteria differentiating NLC from RLS and PLMD. *Percentages; ** if ‘no’ excluded from these survey (*n* = 3)

## Discussion

This review has identified seven criteria, derived from consensus, which can be employed as a framework to differentiate NLC from RLS or PLMD.

Distress and sleep disruption are indicated in a limited number of studies and are associated with negative impacts on physically related aspects of the quality of life [4]. Similarly, ‘subsequent pain’ is also a criterion as a consequence of the occurrence of NLC. The NLC characteristics that were revealed as the most discriminatory – compared to those of RLS and PLMD – include intense pain with duration of a maximum of ten minutes in the calf or the foot, with relief of the symptoms occurring with no intervention. In contrast to NLC, pain in rest or during sleep in the calf or foot can also be due to vascular insufficiency.

In contrast to previous studies, that included all kind of cramps in different ages, the current review focused on NLC among older adults aging 50 years and older therefore excludes other types of cramps [1–3, 28]. Naylor et al. 1994, showed the highest prevalence of NLC is in age group 60-69 years, which is in line with our result with a mean age of the total population in the included studies of 64 years [25]. We also confirmed the previous findings that vascular and renal comorbidities are the most stated and are clinically relevant in elderly people [4, 9, 19–21, 25, 27, 29]. In addition, the use of diuretics is known to cause muscle cramps [9, 16, 21, 24–26, 29]. Consequently, we suggest that vascular and renal comorbidities as well as the use of diuretics could be considered as correlational factors for NLC. This may improve the accuracy of future NLC diagnoses.

Managing the symptoms of patients with NLC can be a challenge in daily clinical practice considering how recent some developments in the diagnosis and treatment of the disorder are. Therefore, we suggest that these developments indicate extending the scope of clinical care. The framework introduced in this review provides a natural guide to future research within the population of older adults with musculoskeletal disorders during rest or sleep. Further research on the reliability and validation of the proposed theoretical framework is necessary for clinical application and diagnostic accuracy.

In addition, two clinical test procedures were reported for diagnostic application: the forceful knee flexion test indicated findings of cramps; the examiner applies a force to overcome knee flexion when testing in a prone position. Most patients with lumbar disc herniation comorbid with leg cramps also showed positive findings during this test, and cramps could be induced (*n* = 2) [26, 30]. There is a need for diagnostic studies in regard to these clinical tests on NLC and it will be interesting to assess their benefits as well [26, 30].

A potential limitation of this review is the lack of primary studies with the focus on diagnosing NLC in older adults. Therefore, as much as possible, the risk of bias was limited by using clearly defined inclusion criteria and conducting a thorough screening and reviewing process of the presented literature. Inherent in this process was the inclusion of patients with several comorbidities in the separate studies. Although these comorbidities might have influenced the interpretation of NLC, it does reflect the clinical relevance of patients with this disorder.

## Conclusions

An extensive history taking including the above seven characteristics may rule out other disorders in diagnosing idiopathic NLC. In conclusion, seven relevant clinical characteristics have been identified to diagnose patients with NLC, and specifically differentiate this disorder from RLS and PLMD. These characteristics enhance the recognition and diagnosis of this highly prevalent, musculoskeletal sleep-related disorder.

## Abbreviations

NLC: Nocturnal leg cramps
PLMD: Periodic limb movement disorder
RLS: Restless leg syndrome
